# Insight from Molecular dynamic simulation of reactive oxygen species in oxidized skin membrane

**DOI:** 10.1038/s41598-018-31609-w

**Published:** 2018-09-05

**Authors:** Surendra Kumar, Dharmendra Kumar Yadav, Eun-Ha Choi, Mi-Hyun Kim

**Affiliations:** 1 Gachon Institute of Pharmaceutical Science & Department of Pharmacy, College of Pharmacy, Gachon University, 191, Hambakmoeiro, Yeonsu-gu, Incheon, 406-799 Korea; 2 Plasma Bioscience Research Center/PDP Research Center, Kwangwoon University, Nowon-Gu, Seoul 139-791 Korea

## Abstract

Non-enzymatic lipid peroxidation of the skin-lipid bilayer causes perturbations that affect the biomembrane structure, function, and permeability of reactive oxygen species (ROS). In the present study, we employed molecular dynamics simulations to study the effect of lipid peroxidation on the bilayer structural properties and permeability of various ROS. The oxidized skin-lipid bilayer was composed of ceramide, cholesterol, free fatty acid, and 5α-hydroperoxycholesterol (5α-CH). The simulation showed that, upon oxidation, the oxidized group (−OOH) of 5α-CH migrates towards the aqueous phase and the backbone of 5α-CH tilts, which causes the membrane to expand laterally. Measurements of the permeability of all ROS along the oxidized skin-lipid bilayer revealed a decreased breaching barrier for all the species as the degree of peroxidation increased, with a resulting easy passage across the membrane. The insights from the simulations indicate that lipid peroxidation might perturb the membrane barrier, thereby inflicting oxidative stress that leads to apoptosis. This study helps to understand oxidative stress at the atomic level. To our knowledge, this is the first reported molecular dynamics simulation study on oxidized skin-lipid bilayer and permeability of ROS.

## Introduction

Skin is the largest organ of the human body is a multilayered structure designed to protect the body against the attack of foreign pathogens, provide a barrier to the permeation of many harmful molecules, and maintain the hydration level of the skin^1,2^. The barrier capacity of the skin attributed to the outermost layer of the skin, termed the stratum corneum, involving the molecular ‘brick and mortar’ organization of corneocytes (brick) and the lipid matrix (mortar)^3,4^. The lipid matrix is the key determinant for the skin’s barrier functions and for the potential non-invasive delivery of therapeutic agents. The lipid matrix compprised of heterogeneous mixture of long-chain ceramides (CER-NS), cholesterol (CHOL), and free fatty acids (FFAs) in an almost equal ratio^5–7^. CER-NS is the most abundant species in the ceramide family and has a sphingosine motif (C18:1) with a fatty acid chain (C24:0). The lipid matrix does contain unsaturated fatty acids, which have an odd number of carbon atoms and branched side chains. However, lignoceric acid (C24:0) is the most abundant FFA found in the stratum corneum^8,9^. CHOL is another important lipid in biological membranes modulates lipid bilayer properties, such as mechanical strength, permeability, fluidity, and phase behavior^10^. The selective inhibition of CER-NS, CHOL, or FFAs can compromise the barrier function of the skin.

Skin lipids are oxidized by three distinct mechanisms of free radical chain oxidation, enzymatic oxidation, and non-radical and non-enzymatic oxidation^11^. In the natural setting, skin lipid undergoes non-enzymatic lipid oxidation, in which photo-oxidation is prevalent and generates various reactive oxygen species (ROS) that include singlet oxygen (^1^O_2_), hydroxyl radical (HO•), and superoxide anion (O_2_^•−^). The role of these ROS in triggering lipid peroxidation has been well documented and they are vulnerable to peroxidative damage to the skin membrane^12–15^. Lipid oxidation is a degenerative process that mainly affects unsaturated lipids, where various types of lipid hydroperoxides form as relatively stable and prominent intermediates^16^. Once these peroxides form, they can accumulate in the lipid bilayer of the skin membrane and further contribute to changes in the structural organization and packing of membrane lipid components. The polyunsaturated fatty acid component of skin membrane lipid is particularly susceptible to peroxidation and can undergo significant changes^17,18^. Among all the lipid compositions, CHOL is an important substrate for peroxidation, which generates hydroperoxycholesterol (ChOOH)^19^. CHOL modulates skin lipid bilayer properties including mechanical strength, permeability, fluidity, and phase behavior, and has been implicated in the dissemination of peroxidative stress^20^. ChOOH includes 5α-ChOOH (5α-CH), 6α-ChOOH, 6β-ChOOH^19^. Furthermore, peroxidation of CHOL also produces Secosterol A, and Secosterol B, an aldehyde product. It has been studied that, Secosterol A and B are present in human plasma and several tissues, but have not yet been reported in the skin lipid membrane^21^. Moreover, 5α-CH is the predominant species found among all the oxidation products and responsible for the structural changes that lead to barrier perturbations and trigger inflammatory responses and carcinogenesis^22^.

The present study investigated the influence of the degree of peroxidation on skin-lipid bilayer membrane properties and permeability of different ROS including hydrogen peroxide (H_2_O_2_), hydroperoxyl (HO_2_•), hydroxyl (HO•), and O_2_ throughout the oxidized skin-lipid bilayer membrane by performing umbrella sampling simulation to measure the potential mean of forces (PMF). The PMF profiles can be used to derive the transfer free energy for permeation across the oxidized lipid bilayer. Neto *et al*. performed the molecular dynamic simulations (MDSs) and studied the effect of phospholipid and CHOL peroxidation on lipid membrane properties^23^. Similarly, Wong-Ekkabut *et al*. performed a MDS of a single component phospholipid bilayer and showed that lipid peroxidation causes membranes to become less densely packed, less ordered, and thinner, which leads to increased permeability^24^. Rai *et al*. performed a multiscale simulation to study the permeability profile of various solutes and drugs on native skin-lipid bilayer membrane^25,26^.

Until now, no MDS study has reported on the oxidized skin-lipid bilayer and the bilayer permeability of ROS. Thus, the present work extends our research to perform constrained MDSs on oxidized skin-lipid bilayer membrane and measure the permeability of ROS across the oxidized skin-lipid bilayer membrane using the umbrella sampling method.

## Materials and Methods

### Description of the model systems

The heterogeneous nature of the stratum corneum lipid layer arises owing to the presence of different CER species comprising varying head groups, FFAs with an odd number of carbon atoms, branched side chains, unsaturated FAs, and CHOL. Our model system only considered the most abundant components of the native skin-lipid bilayer membrane structure CER NS (C24:1), CHOL, and FFA (C24:0).

**Figure 1:**
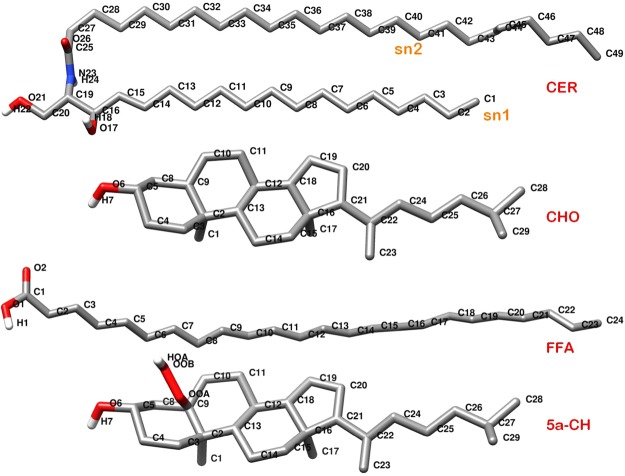
Molecular structure of individual lipids, i.e. Ceramide NS (CER), Cholesterol (CHO), Free fatty acid (FFA) and 5α-ChOOH (5α-CH) used in simulations. Oxygen, Hydrogen, Carbon and Nitrogen atoms are shown in red, white, grey and blue color respectively.

The assembled skin-lipid bilayer structure contained a heterogeneous mixture of 154 molecules, with CER NS (52), CHOL (50), and FFA (52) in an almost equal molar ratio. Together they comprised a non-oxidized (native) skin-lipid bilayer system. CHOL is mainly responsible for maintaining the proper fluidity and rigidity of the lipid bilayer membrane. Thus, lipid peroxidation may decrease the CHOL concentration, which weakens the membrane and makes it more prone to oxidative stress. Oxidative stress increases as the concentration of CHOL in native skin-lipid bilayer membrane falls below 50 mol%^27–30^. To understand the degree of peroxidation on skin-membrane properties, two representative oxidized skin-lipid bilayer systems were constructed by replacing the equivalent of 12.5 and 50 mol% of CHOL with 5α-ChH (a major cholesterol peroxidation product). The individual lipid components are summarized in Fig. 1 and Table 1.

**Table 1:** Composition of the model membranes simulated.

Oxidized Skin-lipid Bilayer System	Composition			
	CER	CHO	FFA	5α-CH
MEMB_12.5 (12.5 mol % of 5α-ChOOH)	52	44	52	6
MEMB_50 (50 mol % of 5α-ChOOH)	52	25	52	25

The total number of lipid molecules was 154 in each bilayer system with 77 in one of the leaflet and 77 in the other.

The oxidized skin-lipid bilayer membrane was constructed using the PACKMOL package^31^. The lipid parameters for CER, CHOL, and FFAs were based on Berger *et al*.^32^ and Holtje *et al*.^33^ For 5α-CH, the parameters were taken from Neto *et al*.^23^ The simple point charge (SPC) model was used for the water molecules^34^. ROS parameters were those reported by Cordeiro^35^. Furthermore the charges and radical nature of ROS were not considered during simulation.

All simulations were performed in the NPT ensemble using the GROMACS 5.1.4 MD package^36–39^. Pressure was controlled at 1 bar by a Parrinello-Rahman barostat with a time constant of 5 ps and compressibility of 4.5 × 10^−5^ bar with semi-isotropic coupling. A time step of 2 fs was used for all simulations. The cut-off distances for Coulombic interactions and van der Waals interactions were set at 1.2 nm. The system was periodic in all Cartesian directions.

The constructed oxidized skin-lipid bilayer was energy minimized using the steepest descent algorithm followed by NVT equilibration for 2 ns under a restrained condition. The equilibrated bilayer was further simulated for a total of 10 ns in the NPT ensemble before the structure was submitted to simulated annealing, where the system was heated to 360 K and cooled to 310.15 K in a systematic manner to obtain well-hydrated lipid bilayer heads. The system was further equilibrated for 50 ns, followed by 200 ns production simulation under NPT ensemble conditions. The final structure of the oxidized skin-lipid bilayer (Fig. S1) was further used to study the various membrane properties that included area per lipid (APL), bilayer thickness, density distribution of individual components, and tail order parameters, and to estimate the free energy profiles (FEPs) of the various ROS across the two different oxidized skin-lipid bilayer systems.

### Analysis of oxidized skin-lipid bilayer membrane properties

To analyze the effect of the degree of lipid peroxidation product, properties of bilayer membrane i.e. the area per lipid (APL), bilayer thickness, density distribution of individual components, and tail order parameters were calculated.

APL is a primary parameter that describes the packing of a lipid bilayer. There are several methods reported for APL calculation^40–42^. However, in the present study, the box-x vector of lipid bilayer was used for the APL calculation with estimates of error during the final 50 ns of the simulation. Similarly, membrane thickness, which is an important parameter that is useful in describing the properties of different oxidized systems, was calculated using APLVORO software^43^ during the final 50 ns of the simulation trajectory. In this analysis, the headgroups of each lipid component (atoms O21 for CER, O6 for CHO, 5α-CH, and O2 for FFA) used as key atoms and membrane thickness was defined as the average position between headgroups of both leaflets. Furthermore, the density distributions of each lipid components were computed to investigate their arrangement along the z-direction. Similarly, the tail order parameters were calculated to characterize the order of lipid hydrocarbons, indicated by the average value of the deuterium order parameter (S_CD_)^44^. It was defined as follows:

$${S}_{CD}=\frac{1}{2}\langle 3{\cos }^{2}({\theta }_{j})-1\rangle $$

where θj is the angle between the C-H bond of the ith carbon and the bilayer normal (z-axis). The angular brackets indicate an ensemble average. An S_CD_ value of 1 means the lipid tails are perfectly oriented along the z-axis, while a value of −0.5 indicates an orientation perpendicular to the bilayer normal axis.

### Permeation free energy profiles (FEPs)

**Figure 2:**
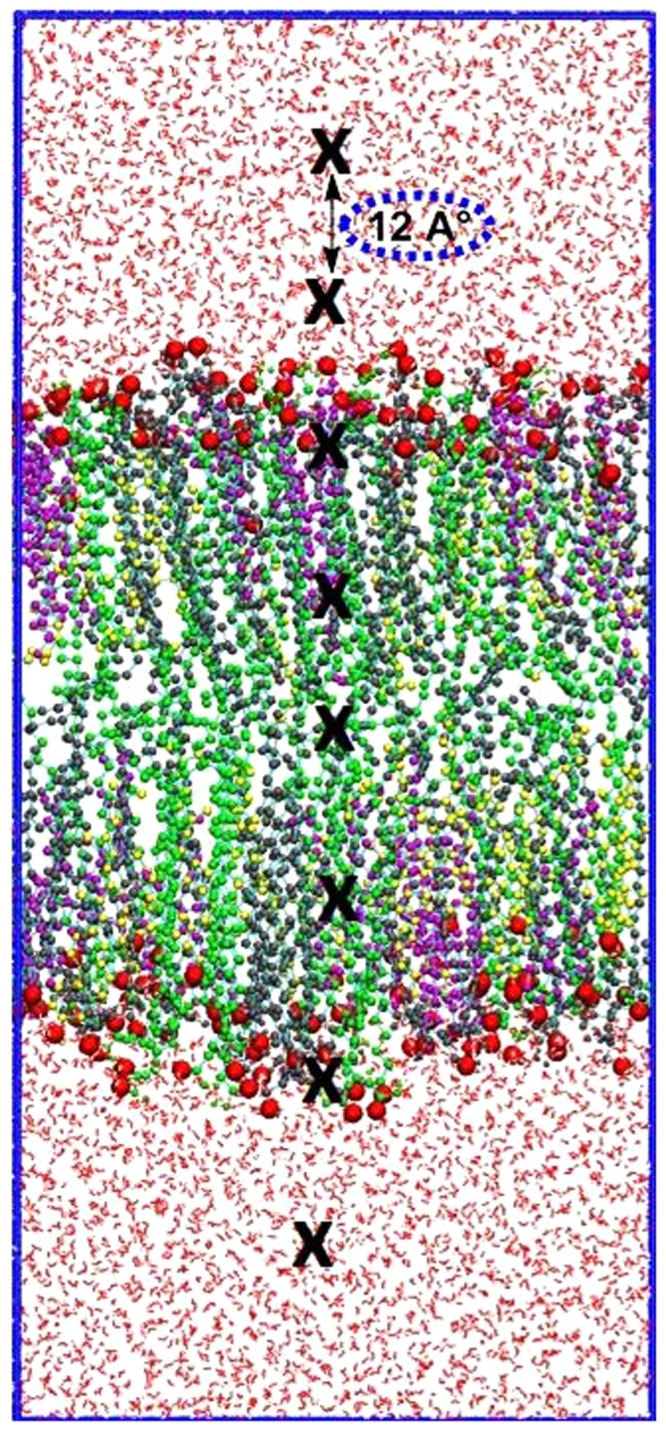
Illustration of the US simulation set-up. Eight umbrella windows separated by 12 Å (position of ROS depicted by block crosses) are sampled in one simulation. In consecutive simulations, each species is shifted by 0.12 Å. To sample the entire membrane system, 33 simulations are performed, yielding a total of 264 umbrella histograms from which a PMF is constructed.

The umbrella sampling simulations, as originally developed^45,46^ were performed to calculate the FEPs of each ROS molecule throughout the oxidized skin-lipid bilayer to explain the 5α-CH concentration-dependent FEP changes. The umbrella simulation method uses an additional energy term (bias) with the system to ensure efficient sampling throughout an entire reaction coordinate^47^. Thus, to sample the whole phase of the membrane bilayer, starting from the upper water leaflet, crossing the lipid bilayer structure, and ending in the lower water leaflet, multiple ROS molecules were placed in the oxidized skin lipid-bilayer system at different xy and z planes. The reaction coordinates of the system were chosen in z dimension, where z = 0 corresponds to the center of mass of the lipid bilayer. To save computational resources, eight umbrella windows were sampled concurrently during each simulation, keeping a distance of 1.2 nm (12 A°) among consecutive windows, starting at approximately 4.8 nm from the center of mass of the bilayer, as illustrated in Fig. 2. For each ROS, a total of 33 systems were created. Each system was energy minimized and equilibrated using the NPT ensemble while keeping the ROS molecules fixed at the current position. Each umbrella simulation lasted for 20 ns, with the last 10 ns used for the analysis, in which the US histograms were acquired and the FEPs calculated. During each umbrella simulation, the ROS molecules were free to move in the xy-plane, but their movement in the z-direction was constrained via applying a harmonic bias with a spring constant of 2000 kJ/mol/nm^-2^. Later, FEPs were constructed using the weighted histogram analysis method, as implemented in GROMACS. To improve the statistical accuracy of sampling, final energy profiles were obtained by averaging six FEPs for each ROS.

## Results and Discussion

### Calculation of oxidized skin-lipid bilayer membrane properties

**Figure 3:**
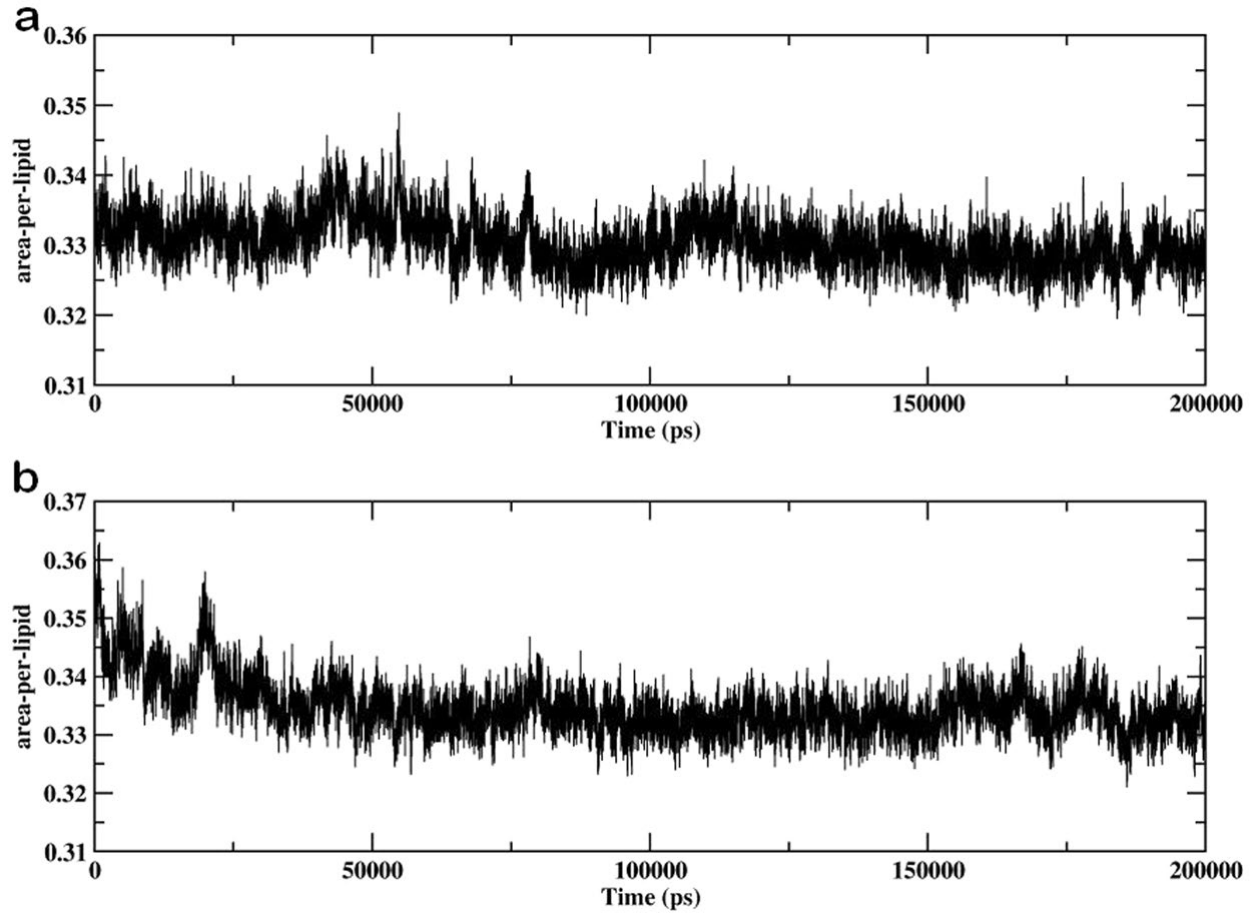
Evolution of area per lipid for (**a**) MEMB_12.5 (**b**) MEMB_50 system.

**Figure 4:**
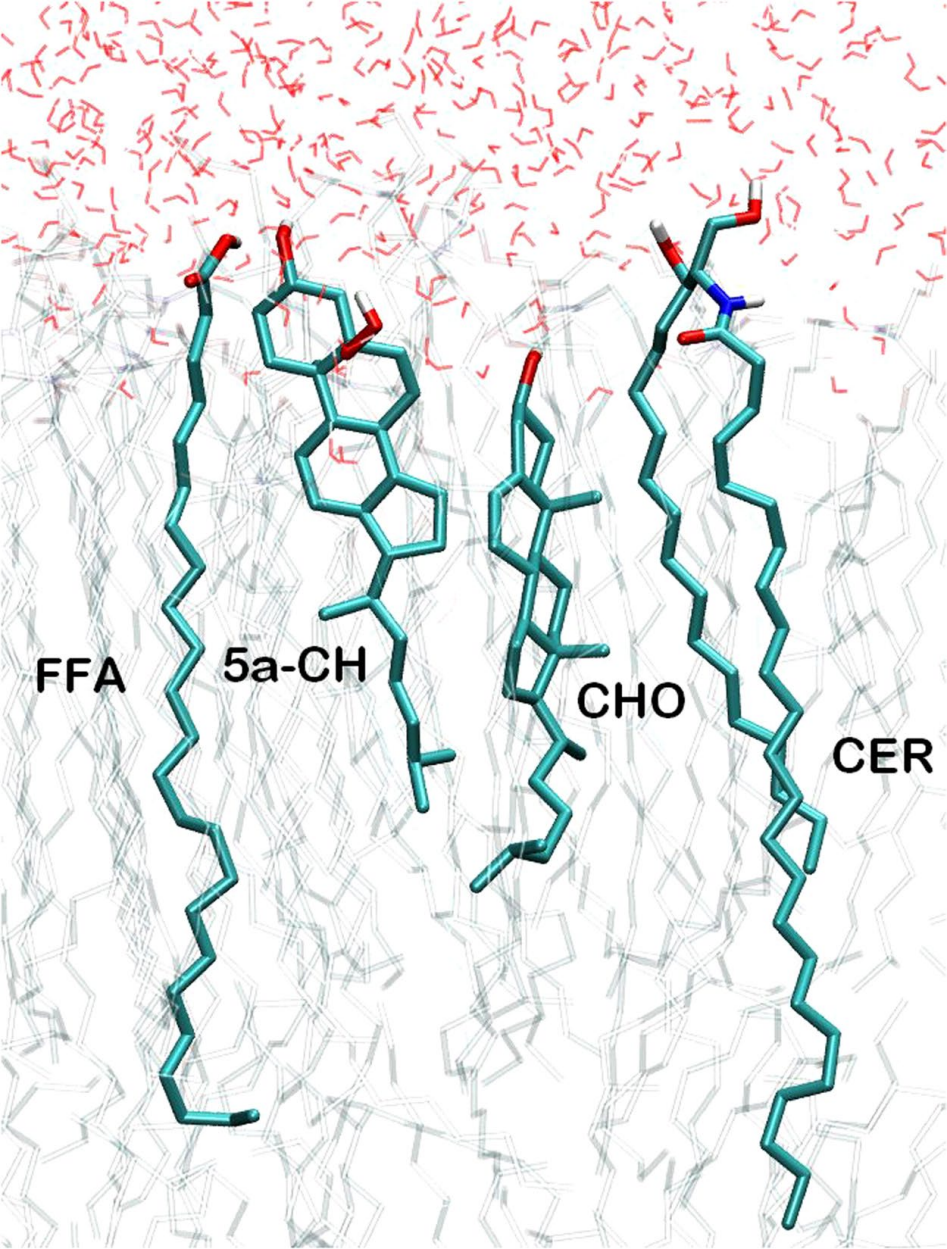
Selected lipids in “CER + CHO + FFA + 5α-CH” systems. Atoms are represented according to the following: White H, cyan C, blue N, and red O.

We investigated the effect of lipid peroxidation products (5α-CH) on the oxidized skin-lipid bilayer membrane properties by calculation of APL. The APLs for oxidized skin-lipid bilayer with 12.5 mol% 5α-CH (MEMB_12.5) and 50 mol% 5α-CH (MEMB_50) were compared. The results are shown in Fig. 3. APLs were 0.328 ± 0.003 nm for MEMB_12.5 (Fig. 3a) and 0.337 ± 0.004 nm for MEMB_50 (Fig. 3b). The higher value for MEMB_50 indicated that upon oxidation the APL for lipid bilayer generally increases. This finding corroborates earlier experimental and computational results^24,48,49^. Upon the oxidation of CHOL components to 5α-CH, the polar groups (-OOH) of the oxidized components preferentially move towards the aqueous layer, to increase their interaction with water molecules (Fig. 4). This bent conformation results in larger APL within oxidized systems.

Similarly, the bilayer thickness of the oxidized skin-lipid bilayer membrane calculated during the last 50 ns simulation was 5.45 nm and 4.90 nm for MEMB_12.5 and MEMB_50, respectively. The measured bilayer thickness decreased with the increase in the oxidized components, and further suggest that the presence of -OOH groups and tilting of the backbone in 5α-CH (an oxidized form of CHOL) occupy extra volume and cause the membrane to expand laterally (Fig. 4). Our result corroborates the prior finding of Neto *et al*.^23^.

**Figure 5:**
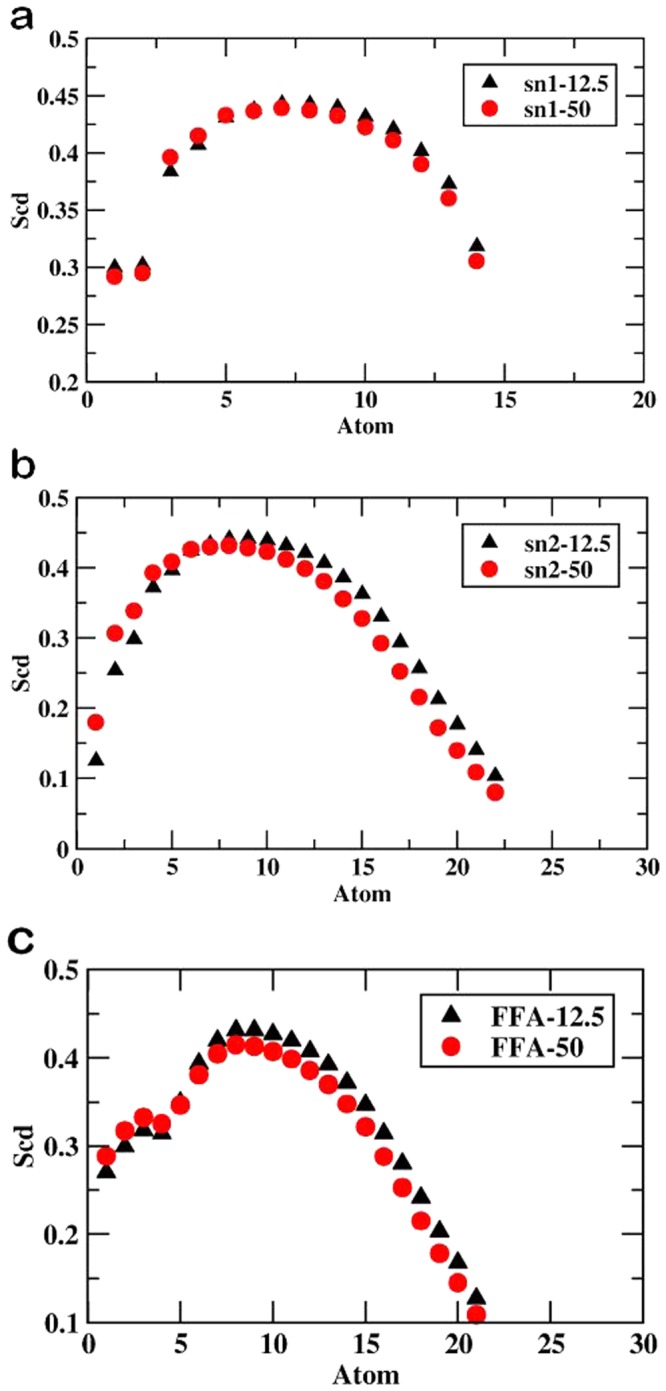
Values of order parameter S_CD_ along the (**a**) sn1 of MEMB_12.5 and MEMB_50; (**b**) sn2 of MEMB_12.5 and MEMB_50 and (**c**) lipid tail of FFA.

Furthermore, the influence of oxidized components over the tail order parameter was calculated for CER and FFA in both oxidized systems. The results are shown in Fig. 5. The order parameters for the sn1 and sn2 tail of CER were low near C16 and C24, respectively, increased towards the mid-bilayer, and decreased further towards the center of the bilayer (Fig. 5a,b). Likewise, in the presence of CER, CHO, and 5α-CH, the order parameters of FFA lipid tails displayed similar trends to the CER chains (Fig. 5c). The tail order profiles for both systems were almost identical. However, the tail order parameters for MEMB_50 were lower as compared to those for MEMB_12.5. An interpretation of the result is that the presence of polar (−OOH) groups of 5α-CH in membrane system distorts the lipid environment, with a more pronounced affect in MEMB_50.

**Figure 6:**
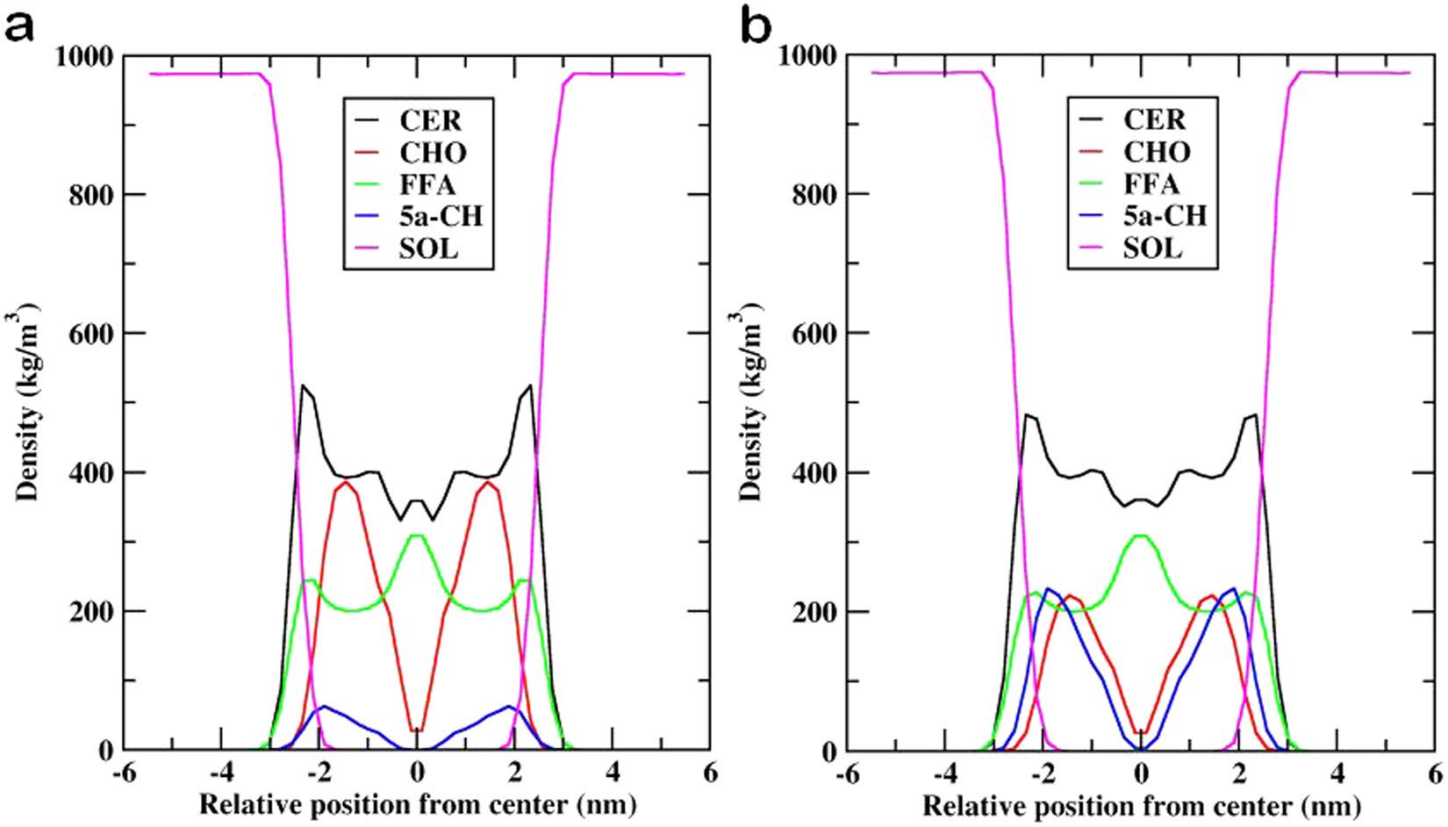
Density profile of individual lipid components (CER-CHO-FFA-5α-CH) for (**a**) MEMB_12.5; (**b**) MEMB_50 along the bilayer normal (z).

Figure 6 depicts a plot of the densities of individual lipid components against the lipid-bilayer normal (z-axis) for both oxidized systems. In both systems, we observed a constant density from z ~ 6.0 nm to z ~ −3.0 nm, which corresponded to bulk water. Further, near the interface, the density for water decreased and headgroups for all the lipids increased (z ~ −3.2 nm to z ~ −1.5 nm). The density for tightly packed lipid tails was found in the region from z ~ −1.5 nm to z ~ −0.4 nm, which is responsible for the skin lipid barrier properties. Lastly, the density was minimal at z ~ 0, which represents the loose and random packing of lipid tails. In Fig. 6, sharp peaks were evident near the lipid-water interface for CER density, with all individual lipid headgroups sitting just below the lipid-water interface. The density profile revealed that CHOL and 5α-CH sit mainly in the high density region of the bilayer due to their smaller size and low partial charge for these headgroups. CER and FFA density profiles only displayed a small peak at the bilayer center that corresponds to proper interdigitation of lipid tails, which is absent for CHOL and 5α-CH due to their small size and shorter chain length.

### Effect of ROS on oxidized skin-lipid bilayer membrane permeability

**Figure 7:**
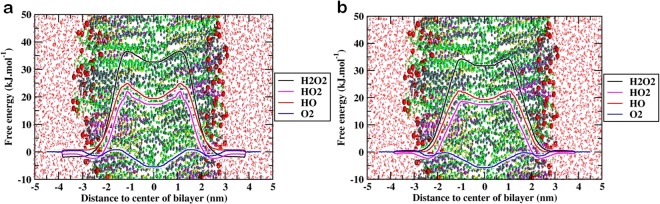
Free energy profile of different reactive oxygen species (ROS) across oxidized skin-lipid bilayer (CER-CHO-FFA-5α-CH) membrane (**a**) MEMB_12.5; (**b**) MEMB_50.

To understand the permeability of various ROS through the two different oxidized skin-lipid bilayer membranes, average PMFs for all ROS were measured. The data are presented in Fig. 7 and Table 2.

**Table 2:** Transfer free energies (ΔG) of all investigated ROS in the oxidized skin-bilayer lipid (CER-CHO-FFA-5α-CH) membrane.

Membrane System	ROS	ΔG(kJ/mol)
MEMB_12.5	H_2_O_2_	37.81 ± 2.05
	HO_2_^•^	24.02 ± 1.62
	HO^•^	25.50 ± 1.73
	O_2_	2.32 ± 0.77
MEMB_50	H_2_O_2_	35.74 ± 1.52
	HO_2_^•^	22.78 ± 1.01
	HO^•^	20.96 ± 1.53
	O_2_	2.29 ± 0.76

### Permeability profile of ROS in the MEMB_12.5 system

The transfer free energy of all ROS along the oxidized skin-lipid bilayer is shown in Fig. 7(a). The membrane shown in the background depicts the location of the different regions of the bilayer structure. The free energy barrier for H_2_O_2_, HO_2_^•^, HO^•^, and O_2_ were low and almost negligible in the aqueous phase. As the ROS approached the headgroups of the lipid bilayer (z ~ 3 nm), the free energy for the H_2_O_2_, HO_2_^•^, and HO^•^ species decreased significantly. This corresponded to the interaction of partially charged hydrophilic headgroups of the bilayer. For O_2_, a slight increase in free energy was observed. These observations are consistent with those presented by Rai *et al*.^25^, where the transfer of free energy of hydrophilic species slightly decreased and, for hydrophobic species, slightly increased. The HO_2_^•^ species displays greater interaction as compared to HO^•^ species in headgroups region. Due to with additional oxygen atom, HO_2_^•^ species has greater van der Waal interactions with headgroups of the lipid bilayer which might explain its deep free energy minima^35^. Furthermore, HO_2_^•^ species are better proton donors but weaker acceptors whereas HO^•^ species act equally as H-bond donor or acceptors than water^35,50^, thus they are more likely to interact with lipid headgroups. These observations agreed with the experimental data and corresponds to the permeability of the different ROS^51^. After crossing the headgroups region of the lipid bilayer, the role of the lipid bilayer membrane as a permeation barrier can be explained on the basis of the drastic increase in free energy for all ROS, except O_2_. This is consistent with experimental study, where H_2_O_2_, HO_2_^•^ and HO^•^ are found to be less permeant than O_2_^52^. The transfer free energy increased and reached a maximum in the hydrophobic core for H_2_O_2_ (~36.35 kJ/mol), HO_2_^•^ (~22.16 kJ/mol), and HO^•^ (~24.77 kJ/mol) and then respectively decreased to ~32.42 kJ/mol, ~17.47 kJ/mol, and ~19.22 kJ/mol at the center of the bilayer. Due to hydrophobic nature of O_2_, its PMF profile was entirely different from the other ROS. The transfer free energy of O_2_ after crossing the headgroups of the lipid bilayer decreased to ~−1.64 kJ/mol and then increased to ~0.69 kJ/mol. This new energy barrier observed at ~1.5 nm for O_2_ species can be assigned to the bulky ring of CHOL and 5α-CH in the skin-lipid bilayer membrane. The transfer free energy for O_2_ species further decreased (~−5.62 kJ/mol) and displayed an energy minima at the center of the bilayer. The free energy increased due to loss of favorable electrostatic interactions and hydrogen bond from the aqueous phase to the hydrophobic phase. In the center of the bilayer, due to lower lipid density, the free energy was decreased^53^. The average of the transfer free energy from 33 independent simulations is presented in Table 2. Comparing the transfer free energy barriers for all ROS revealed that the permeation of H_2_O_2_ was most hindered at an estimated free energy of 37.81 ± 2.05 kJ/mol, while O_2_ was least hindered at an estimated free energy of 2.32 ± 0.77 kJ/mol.

### Permeability profile of ROS in the MEMB_50 system

Figure 7(b) depicts the transfer free energy results for ROS along the oxidized skin-lipid bilayer membrane. The PMF profile was similar to the PMF profile of MEMB_12.5. As the ROS reached the headgroups of the lipid bilayer (z ~3), the transfer free energy decreased and, as the ROS entered the hydrophobic region of the lipid bilayer, the transfer free energy increased and maximal values were found in the high-density region of the oxidized skin-lipid bilayer. With further movement, the transfer free energy decreased. We observed the maximal transfer free energy for H_2_O_2_ (~34.45 kJ/mol), HO_2_^•^ (~18.91 kJ/mol), HO^•^ (~22.45 kJ/mol), O_2_ (~ 0.65 kJ/mol) and respectively minimal energies of ~31.89 kJ/mol, ~17.62 kJ/mol, ~19.24 kJ/mol, and ~ −5.81 kJ/mol. The average transfer free energies of 33 independent simulation results are shown in Table 2. Comparing the transfer free energy barriers for all ROS on MEMB_50 system revealed that H_2_O_2_ was most hindered with a transfer free energy of 35.74 ± 1.52 kJ/mol, while O_2_ was least hindered with a value of 2.29 ± 0.76 kJ/mol.

The MEMB_12.5 and MEMB_50 oxidized skin-lipid bilayer systems differ in the number of oxidized components (5α-ChH). Upon oxidation of the skin-lipid bilayers, the APL increased and the membrane bilayer thickness decreased, while the membrane expanded laterally. These behaviors correspond to the accommodation of polar groups (−OOH) of 5α-CH in the headgroups region of the lipid bilayer. Such structural changes in the lipid bilayer membrane cause the system to become less densely packed, thinner, and less ordered, which makes the system more vulnerable to ROS. The PMF profiles of all ROS in the oxidized systems showed similar trends to the work of Van der Paal *et al*.^54^, in which different phospholipid membrane systems consisting of various concentration of CHOL (0 to 50 mol%) were built and permeability of various ROS was measured. Moreover, comparing the transfer free energy values of all ROS for the MEMB_12.5 and MEMB_50 systems revealed that increasing the oxidized components in skin-lipid bilayers reduced the resistance properties of the membranes and decreased the transfer free energy values. Thus, ROS could easily breach the free energy barriers and permeate easily across the membrane, causing oxidative stress that might lead to apoptosis.

## Conclusion

Lipid peroxidation of the skin-lipid bilayer contributes to altered structural organization, such as lipid packing, thermodynamics, and phase parameters of membranes. The structural changes caused by lipid peroxidation lead to skin-lipid barrier perturbations, inflammatory responses, and carcinogenesis. In the present study, the effect of concentration-dependent 5α-CH (a major lipid-peroxidation product) on membrane structural properties and permeation of various ROS across the oxidized skin-lipid bilayers was studied using molecular dynamic simulations. We modeled two oxidized skin-lipid bilayer membrane systems. MEMB_12.5 and MEMB_50 were comprised of CER, CHOL, FFA, and 5α-CH. The membrane properties that were studied included APL, bilayer thickness, density profile of individual lipid components, and tail order properties. The results revealed that, upon increasing the concentration of 5α-CH, the APL increased and membrane bilayer thickness decreased. During the simulations, polar groups (−OOH) of 5α-CH tilted and oriented toward the aqueous phase along the bilayer normal axis, causing the bilayer membrane to expand laterally and disrupt the structural integrity of the membrane. We further investigated the permeability of ROS across these oxidized skin-lipid bilayer membranes. The transfer free energy in terms of the potential mean of force for MEMB_12.5 and MEMB_50 indicated that free energy barrier for all reactive oxygen species decreased as the concentration of the peroxidized lipid component increased. Likewise, the minimal free energy barrier observed for MEMB_50 as compared to MEMB_12.5 suggested that lipid peroxidation of skin-lipid bilayer membrane has detrimental effects on barrier properties. Thus, lipid peroxidation perturbs skin-lipid bilayer membranes, which facilitates the easy movement of ROS along the lipid bilayer. By breaching the free energy barrier at low transfer energy, all ROS would be able to permeate across the membrane to reach the interior, causing oxidative stress that might lead to apoptosis. In conclusion, our results indicate that increased lipid peroxidation of skin-lipid membranes impairs the barrier function, which lowers the transfer free energy for ROS.

## Electronic supplementary material

Supplementary Dataset 1
