# Supplementary material for: Insight from Molecular dynamic simulation of reactive oxygen species in oxidized skin membrane
Source: Sci Rep. 2018 Sep 5;8:13271. doi: 10.1038/s41598-018-31609-w (PMC6125594; doi:10.1038/s41598-018-31609-w)

**SUPPLEMENTARY DATA**

**Insight from Molecular Dynamic Simulation of Reactive Oxygen Species In Oxidized Skin Membrane**

Surendra Kumar^1#^, Dharmendra Kumar Yadav^1#,^*_,_ Eun-Ha Choi^2^, Mi-Hyun Kim^1,*^

^1^College of Pharmacy, Gachon University of Medicine and Science, Hambakmoeiro, 191, Yeonsu-gu, Incheon, 406-799, Korea

^2^Plasma Bioscience Research Center/PDP Research Center, Kwangwoon University, Nowon-Gu, Seoul 139-791, Korea

*E.mail:* [*dharmendra30oct@gmail.com*](mailto:dharmendra30oct@gmail.com)*,* [*kmh0515@gachon.ac.kr*](mailto:kmh0515@gachon.ac.kr)

^#^These authors contributed equally to the work

*corresponding author

**Dr. Dharmendra Kumar Yadav, Ph.D**

Research Assistant Professor

Office: +82-32-820-4947

Email: [dharmendra30oct@gmail.com](mailto:dharmendra30oct@gmail.com)

**Dr. Mi-hyun Kim, Ph.D**

Assistant Professor

Office: +82-32-820-4947

Email: [mhmjmj@gmail.com](mailto:mhmjmj@gmail.com)

**Figure S1: Oxidized skin-lipid bilayer sstructure containing a) 52 CER (Grey), 44 CHO (Yellow), 52 FFA (Green), 6 5α-CH (Magenta) molecules and 5210 water molecules; b) 52 CER (Grey), 25 CHO (Purple), 52 FFA (Yellow), 25 5α-CH (Magenta) molecules and 5210 water molecules. Headgroups are shown in pink sphere (VDW).**


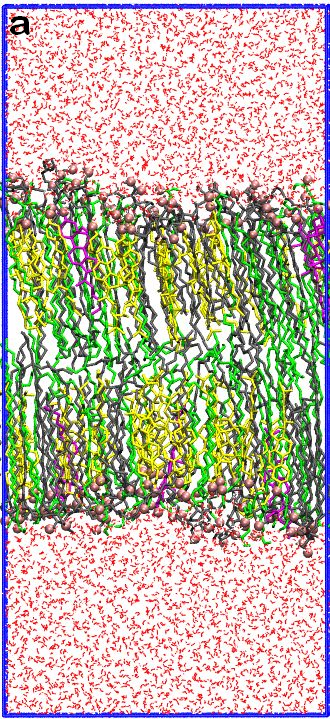

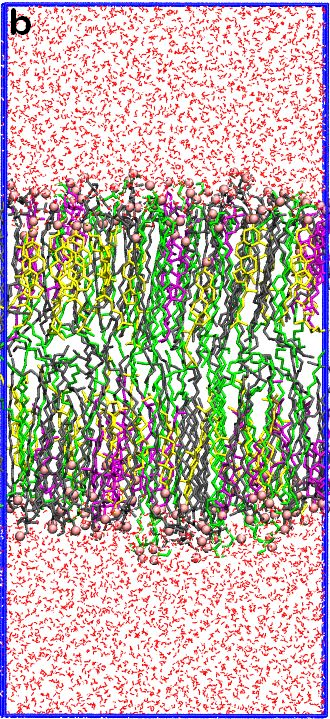

Supplement: Supplementary file 1 — Supplementary Dataset 1 [file 41598_2018_31609_MOESM1_ESM.docx]
